# Brain Surgery

**Published:** 1895-10-05

**Authors:** 


### PROGRESS IN SURGERY.

### BRAIN SURGERY.

(*Continued from page 449, Vol. XVIII.*)

**Operations for Cerebral Tumours.**—In his exhaustive work on the operative surgery of the nervous system, Chipault[25] speaks hopefully of operations for cerebral new growths. Out of sixty-seven cases of tumours removed tabulated by him, there were forty-seven recoveries (70 per cent.). Scarcely less striking is his statement, based on pathological investigations, that only from 6 to 10 per cent. of cerebral tumours are removable by operation. In the light of this statement, we are not astonished to find that in cases where operations have been performed, and either no tumour has been detected or, if detected, has been impossible of removal, the mortality is 75 per cent.

A number of fresh examples have recently been recorded. Cotterill[6] removed a large tubercular tumour involving the frontal bone, the frontal sinus, and at some places the dura mater. The greater part of the frontal bone was removed, and it was replaced by a celluloid plate. The patient made a rapid recovery.

Wood[26] reports a very successful operation for the removal of a sub-cortical glioma growing from the arm centre in the cerebrum. Although the tumour recurred four months later, it was again attacked and removed, and seven months after the second operation the patient remained quite well. Dana[27] operated on a youth for a spindle celled sarcoma, which appears to have had its primary seat in the dura, but which came to involve the left arm and finger centre, and to produce all the classic symptoms of brain tumour. Unfortunately, it was found impossible to remove all the growth, but in spite of this, for the last six months he has been in fairly good health. In another case, Bremer and Carson[28] failed to completely remove a large tumour of the dura mater (cylindroma endotheliodes) from a patient aged forty-six, who died on the seventeenth day. Morton[29] operated for Dr. Mitchell Bruce on a patient who presented all the classical symptoms of cerebral tumour—apparently of great size. The operation was done simply to relieve intra-cranial tension, and was successful to the extent that the man recovered his mental powers, and was enabled to lead a comfortable existence for at least six months after the operation.

**Dermoid Cyst.**—A case of pathological as well as clinical interest is reported by Walther,[30] who claims it to be the only recorded case of its kind. It was a dermoid cyst, which was partly inside the skull and partly outside, the two parts being connected by a small aperture. This case, Walther holds, gives great support to the inclusion theory of dermoid cysts, a piece of the ecto-derm having been imperfectly cut off by the developing cranial bones. Clinically the interest of the case lay in the fact that the cyst gave rise to all the symptoms of a cerebral tumour, which were removed by free opening and drainage of the cyst.

**Optic Neuritis in Cerebral Tumours.**—Proof that optic neuritis in cases of cerebral tumour is due to great increase in the intra-cranial tension is afforded by the observations of Taylor.[31] He finds that the signs of optic neuritis disappear after opening the skull, whether the tumour be removed or not. Horsley first pointed this out.

**Tapping the Vertebral Canal.**—It may be admitted that operative measures employed for the confirmation of a diagnosis, which may be rendered fairly certain on ordinary clinical grounds, are only to be employed if they be devoid of risk in themselves. Of such a disputable nature is the operation of tapping the vertebral canal in supposed cases of tubercular meningitis. This procedure was the subject of discussion at a recent meeting of the Berlin Medical Society.[22] Fürbringer spoke from an experience of 100 tappings. The operation he finds easy, and chloroform is unnecessary. He places the patient in a sitting posture, and taps through the 2nd, 3rd, or 4th lumbar interspace. Fraenkel makes the puncture near the junction of the upper with the middle third of the spinous process selected, and about two fingers' breadths from the middle line. After piercing the skin the needle is turned upwards and inwards.

In tubercular meningitis Fürbringer has employed the method 37 times, and in 30 the tubercle bacillus was found in the fluid. Fraenkel found the bacillus in some cases only, and failed to find it in others undoubtedly tubercular. Heubner failed to find it in all his six or seven cases. Freyhan believes that the proportionate amount of albumen found in the fluid is of diagnostic significance. In simple meningitis it is much richer in albumen than in the tubercular variety.

A diagnosis of epidemic cerebro-spinal meningitis in one case, and of acute purulent meningitis in another, has been made by Fürbringer by means of the fluid withdrawn. The same observer has diagnosed in two cases of apoplexy that the ventricles were ruptured by finding blood in the fluid. Freyhan adds that unimpeachable evidence of laceration of the ventricles is furnished by the fluid containing a large quantity of blood. In meningeal hæmorrhage it is only slightly tinted with blood. In cases of spinal tumour the diagnostic results of tapping have been negative.

The therapeutic value of tapping is doubtful. Relief has been given in many cases where the cerebro-spinal tension has been very high, but it has been ephemeral, and in only one of Fürbringer's cases (chronic serous meningitis) has it been lasting. One case of Fraenkel's was apparently cured and one relieved by the measure. Ord and Waterhouse[23] had a successful case of tubercular meningitis. In cases of chronic hydrocephalus Heubner prefers lumbar to cranial puncture for the relief of tension.

[22] Med. Week, Mar. 20, 1895. (Berl. Klin. Woch, Ap., 1895.) [23] Lancet, 1895. [24] Clin. Journ., July 17, 1895. [25] Chirurg. Operat. du Sys. Nerv., 1895. [26] N.Y. Med. Journ., Mar. 23, 1895. [27] Med. Week, Ap. 19, 1895. [28] Amer. Journ. Med. Science, Feb. 1895. [29] B.M.J., Ap. 13, 1895. [30] Presse Médicale, Ap. 1895. [31] Trans. Ophthal. Soc., Vol. XIV.

